# The impact of college students’ dual filial piety on filial practice: the mediating roles of filial perception and parent–child relationship

**DOI:** 10.1186/s40359-026-04954-z

**Published:** 2026-07-07

**Authors:** Yizhan Du, Zengming Ge

**Affiliations:** 1 School of Education, Fujian Polytechnic Normal University, Fuqing, 350300 China; 2 School of Psychology, Shinawatra University, Bangkok, 12160 Thailand

**Keywords:** Dual filial piety, Filial practice, Filial perception, Parent–child relationship

## Abstract

**Background:**

In the context of social transformation and the reconstruction of intergenerational ethics, this study examined whether reciprocal filial piety and authoritarian filial piety were associated with college students’ filial practice through filial perception and parent–child relationship.

**Methods:**

A cross-sectional questionnaire survey was conducted among 821 college students in Guizhou Province, China, using the Dual Filial Piety Scale, Parent–Child Relationship Scale, Filial Perception Scale, and Filial Practice Scale. Descriptive statistics, correlation analysis, regression analysis, bootstrapped mediation analysis, and structural equation modeling were used to examine the proposed associations.

**Results:**

Reciprocal filial piety was positively associated with filial practice indirectly through parent–child relationship and filial perception. Authoritarian filial piety was negatively associated with filial practice indirectly through parent–child relationship, whereas its indirect path through filial perception was not statistically significant. In both models, the direct effects of filial piety on filial practice were not significant.

**Conclusions:**

The findings provide cross-sectional evidence that dual filial piety is related to filial practice mainly through relational and cognitive pathways. Reciprocal filial piety was linked to more favorable parent–child relationship quality and stronger filial perception, whereas authoritarian filial piety was linked primarily to poorer parent–child relationship. These findings should be interpreted cautiously because the study used a cross-sectional design and a regionally bounded convenience sample.

## Introduction

Throughout the thousand-year-long ethical genealogy of Chinese civilization, "filial piety" has always been the core value criterion running through intergenerational relationships [1, 2]. In "The Book of Rites・The Meaning of Sacrifices", the classic statement "The greatest filial piety is to respect one's parents, the second is not to disgrace them, and the lowest is to be able to support them" constructs a multi-layered system of filial piety from spiritual respect to behavioral practice. It contains not only the cultural genes of intergenerational emotional connection but also the ethical norms of responsibility fulfillment [3, 4]. As a theoretical framework for deconstructing traditional filial piety in the modern context, the dual filial piety model [5, 6] divides filial piety into reciprocal filial piety (emphasizing equal interaction between parents and children) and authoritarian filial piety (focusing on children's submissive responsibility). Together, they shape the essential characteristics of intergenerational relationships [7–9]. Among them, reciprocal filial piety embodies the modern intergenerational logic of emotional resonance and two-way support, while authoritarian filial piety carries the traditional ethical connotations of respecting authority and fulfilling obligations [10, 11]. In-depth exploration of the relationship between dual filial piety and filial practices has important practical significance. On the one hand, it helps to deepen the theoretical understanding of family intergenerational ethics and provide theoretical support for the transformation of modern family relationships [12, 13]. On the other hand, it can provide guidance for contemporary family education practice and the harmonious development of society, and facilitate the modern transformation of traditional filial piety culture [14]. Although existing studies have paid attention to the impact of dual filial piety on individual psychology and behavior [5, 8, 15], research on filial practices, a core behavioral manifestation, still has limitations. Most of the research remains at the analyzing direct effects of a single dimension of filial piety. There is no systematic empirical conclusion regarding the internal mechanisms by which dual filial piety affects filial practices, especially the mediating roles of filial perception and parent–child relationships. Therefore, this study will start from a dual perspective to comprehensively examine the influence path and psychological mechanisms of dual filial piety on filial practices.

Previous studies suggest that, during emerging adulthood, filial practice is shaped not only by filial values themselves but also by how young adults understand their responsibilities and experience their relationships with parents [16–18]. Reciprocal filial piety emphasizes mutual care and respect, whereas authoritarian filial piety emphasizes obedience and hierarchical obligation. These two dimensions may therefore relate to filial practice through different mechanisms [19–21]. In particular, filial perception reflects how clearly and willingly individuals recognize children’s responsibilities toward parents, while parent–child relationship quality reflects the emotional and interactional context in which those responsibilities are enacted [22–25]. Existing studies have rarely examined these two pathways within the same framework among college students in contemporary China [26].

**Fig. 1:**
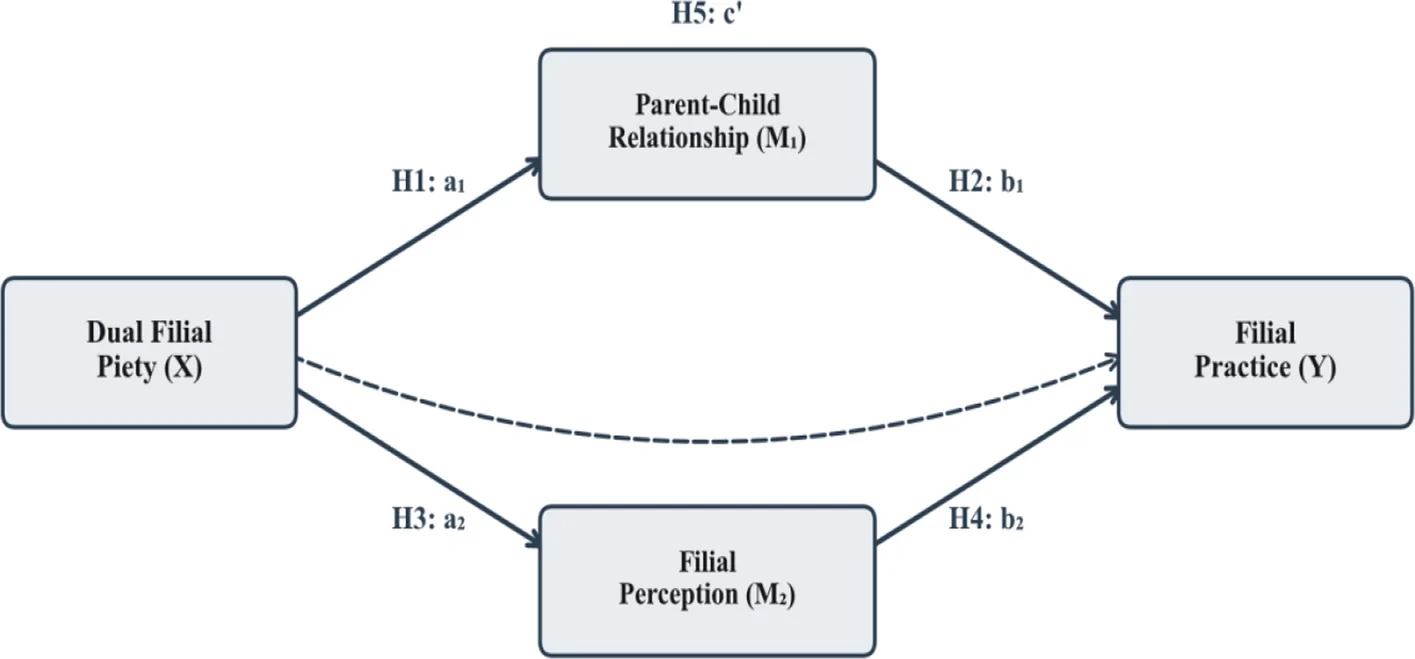
Hypothesized parallel mediation framework tested separately for reciprocal and authoritarian filial piety

**Fig. 2:**
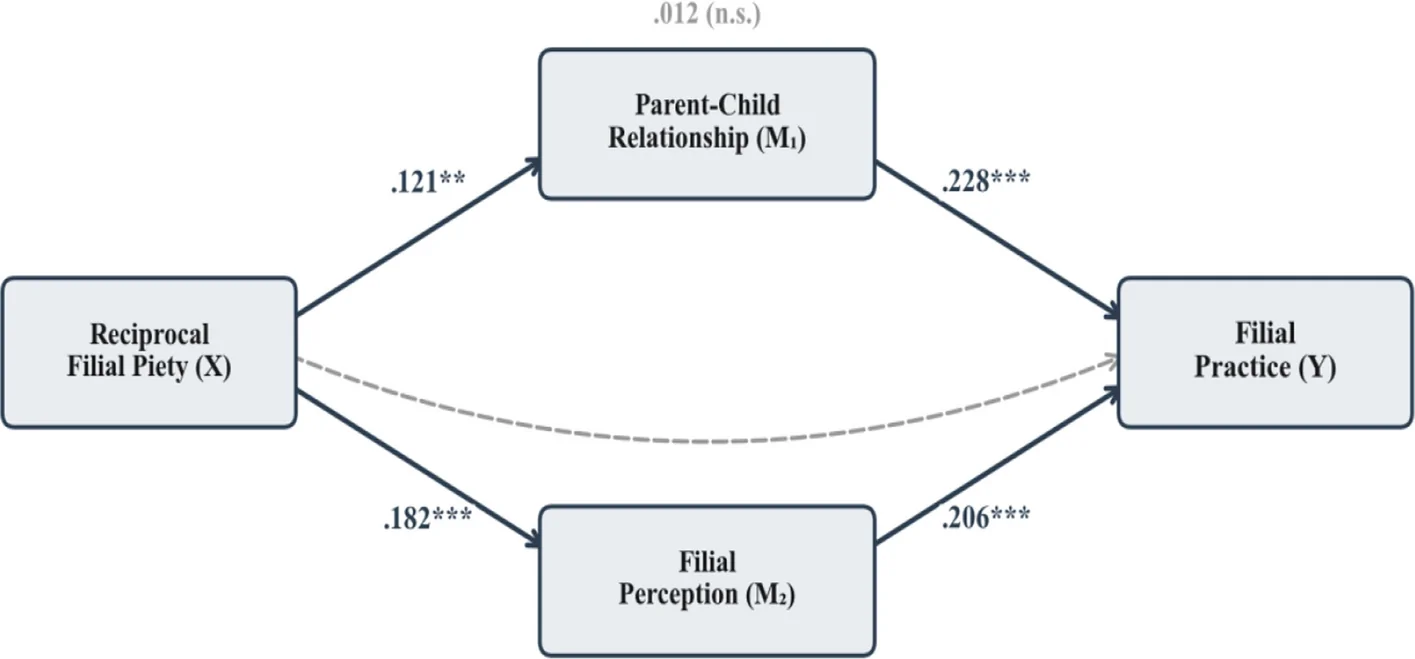
Estimated mediation model for reciprocal filial piety

**Fig. 3:**
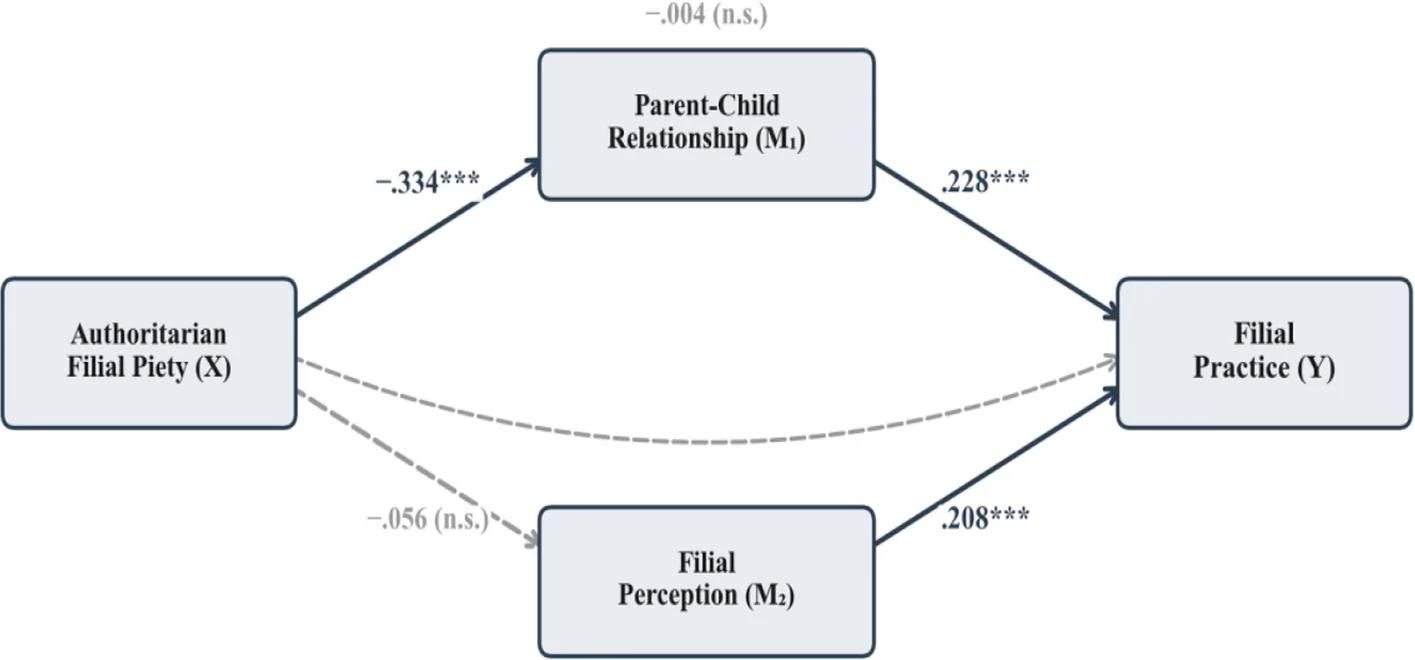
Estimated mediation model for authoritarian filial piety

Accordingly, this study tested a parallel mediation framework in which filial perception and parent–child relationship were specified as two mediators between filial piety and filial practice. Because reciprocal filial piety and authoritarian filial piety are conceptually distinct dimensions, we examined the same mediation structure separately for each dimension. Fig. 1 presents the hypothesized framework, and Figs. 2 and 3 present the estimated models for reciprocal filial piety and authoritarian filial piety, respectively.

Based on this framework, four hypotheses were proposed. H1: Reciprocal filial piety is positively associated with filial practice through parent–child relationship. H2: Authoritarian filial piety is negatively associated with filial practice through parent–child relationship. H3: Reciprocal filial piety is positively associated with filial practice through filial perception. H4: Authoritarian filial piety is negatively associated with filial practice through filial perception.

## Research methods

### Subjects

Using convenience sampling, college students in Guizhou Province, China, were recruited through group-administered questionnaire sessions coordinated by the research team. Participation was voluntary. Before participation, all respondents were informed of the purpose and procedures of the study, provided written informed consent, and were told that they could withdraw at any time without penalty. Data were collected in April 2025. After routine data screening for completeness and response quality, 821 questionnaires were retained as valid cases for analysis. The study protocol was approved by the Ethics Committee of Fujian Polytechnic Normal University (Approval No. 2025–05). Among them, 364 were male students (44.30%), and 457 were female students (55.70%). In terms of academic years, 254 were freshmen (30.90%), 237 were sophomores (28.90%), 162 were juniors (19.70%), and 168 were seniors (20.50%).

### Research instruments

#### Dual-filial-piety scale

In this study, the revised Dual-Filial-Piety Scale [27, 28] was adopted. This scale is suitable for subjects in Mainland China. It consists of two dimensions: authoritarian filial piety (e.g., "When you disagree with your parents, you should follow their opinions." There are 8 such items in total) and reciprocal filial piety (e.g., "Pay more attention to your parents' physical health." There are 8 such items in total), with a total of 16 items. A 6-point rating scale was used (1 = strongly disagree, 6 = strongly agree). The average scores of the 8 items in each of the two dimensions were calculated separately. The higher the score, the higher the individual's level of filial piety. In this study, the internal consistency reliability (Cronbach’s *α*) of the scale was as follows: for reciprocal filial piety, *α* = 0.85; for authoritarian filial piety, *α* = 0.91, indicating that the scale has good reliability. Convergent validity: Reciprocal filial piety (AVE = 0.42, CR = 0.85), Authoritarian filial piety (AVE = 0.56, CR = 0.91). Though AVE < 0.5 for reciprocal dimension, CR > 0.7 and factor loadings > 0.6 support construct reliability.

#### Parent–child relationship scale

In this study, a scale adapted from the Parental Attachment Scale [29, 30] was used to assess parent–child relationship quality. It contains 12 items and three subscales: Communication (e.g., “I tell my parents about my problems and troubles”; 4 items), Trust (e.g., “My parents respect my feelings”; 4 items), and Alienation (e.g., “I feel angry with my parents”; 4 items). Items were rated on a 5-point Likert scale ranging from 1= completely disagree to 5 = completely agree. After reverse scoring the alienation items, higher scores indicated better parent–child relationship quality. In this study, the Cronbach’s α coefficient for the total scale was 0.747, and the coefficients for the Communication, Trust, and Alienation subscales were 0.652, 0.782, and 0.860, respectively. The construct validity indices were χ2/df = 3.508, CFI = 0.975, TLI = 0.968, RMSEA = 0.055, and SRMR = 0.036.

#### Filial perception scale

In this study, the Filial Perception Scale [31, 32] was employed. It contains a total of 18 items and is divided into three sub-scales: Life Care Dimension: (e.g., At home, I feel that I don't get enough attention; 7 items in total). Emotional Comfort Dimension: (e.g., When I have conflicts with my parents, I should communicate with them calmly. There are 7 such items). Growth and Development Dimension: (e.g., If my parents do something inappropriate, I should persuade them and explain the reasons. There are 4 such items). A 5-point scoring system is used (1 = completely inconsistent, 5 = completely consistent). The internal consistency reliability (Cronbach’s *α*) of the scale is 0.83. Specifically, for the Life Care Dimension, *α* = 0.83; for the Emotional Comfort Dimension, *α* = 0.85; and for the Growth and Development Dimension, *α* = 0.77. The convergent validity, with AVE and CR being 0.43 and 0.93 respectively [33].

#### Filial practice scale

In this study, the Filial Practice Scale [32, 34] was adopted. It consists of 19 items and is divided into three sub-scales: Life Care Dimension: (e.g., I can reasonably plan my monthly living expenses. There are 4 such items). Emotional Comfort Dimension: (e.g., I know what my parents' favorite food is. There are 8 such items). Growth and Development Dimension: (e.g., Every time my parents point out my shortcomings, I will humbly correct them. There are 7 such items). A 5-point scoring system is used (1 = completely inconsistent, 5 = completely consistent). The internal consistency reliability (Cronbach’s *α*) of the scale is 0.81. Specifically, for the Life Care Dimension, *α* = 0.82; for the Emotional Comfort Dimension, *α* = 0.85; and for the Growth and Development Dimension, *α* = 0.87. The convergent validity, with AVE and CR being 0.47 and 0.94 respectively [15].

### Data processing and statistical analysis

Descriptive statistics and correlation analyses were conducted in SPSS 26.0. Confirmatory factor analyses and structural equation models were estimated in AMOS 26.0. Bootstrapped mediation analyses (5,000 resamples) were conducted in PROCESS 4.1. To align the statistical analyses with the conceptual model, two parallel mediation models were tested separately: one with reciprocal filial piety as the predictor and one with authoritarian filial piety as the predictor; in both models, filial perception and parent–child relationship were specified as mediators and filial practice as the outcome.

## Results

### Common method bias test

Statistically, the single common method factor control approach was adopted for the test. The results of confirmatory factor analysis showed that the single-factor model had a poor fit: χ2/df = 9.43, CFI = 0.13, NFI = 0.12, RMSEA = 0.10, and AGFI = 0.34. This indicates that common method bias was not a serious concern in this study.

### Descriptive statistics and correlation analysis of variables

The means (M), standard deviations (SD), and Pearson product–moment correlation coefficients of the main variables are presented in Table 1. Reciprocal filial piety was positively correlated with parent–child relationship, filial perception, and filial practice, whereas authoritarian filial piety was negatively correlated with parent–child relationship and filial practice and was not significantly correlated with filial perception. Parent–child relationship and filial perception were both positively correlated with filial practice.

**Table 1:** Descriptive statistics and correlations among the main variables (*N* = 821)

	**M**	**SD**	**1**	**2**	**3**	**4**	**5**
1. Reciprocal filial piety	4.723	0.695					
2. Authoritarian filial piety	3.828	0.881	-.653**				
3. Parent–child relationship	2.633	0.529	.121**	-.334**			
4. Filial perception	3.845	0.494	.182**	−0.056	0.054		
5. Filial practices	3.852	0.463	.077*	-.092**	.241**	.221**	

^*^p <.05; **p <.01

### Regression analysis

According to the regression analysis results in Table 2, neither authoritarian filial piety (β = 0.016, *p* > 0.05) nor reciprocal filial piety (β = 0.008, *p* > 0.05) showed a statistically significant direct association with filial practice after the mediators were entered into the model. In contrast, parent–child relationship (β = 0.230, *p* < 0.001) and filial perception (β = 0.206, *p* < 0.001) were positively associated with filial practice. All VIF values were below 2, indicating no serious multicollinearity.

**Table 2:** Multiple regression analysis predicting filial practice (*N* = 821)

	**b**	**SE**	**β**	**t**	**p**	**95% CI**		**Tolerance**	**VIF**
(Constant)	2.515	0.258		9.74	<.001	2.008	3.022		
Reciprocal Filial Piety	0.011	0.030	0.016	0.365	0.715	−0.048	0.07	0.541	1.85
Authoritarian filial piety	0.004	0.025	0.008	0.161	0.872	−0.044	0.052	0.502	1.991
parent–child Relationship	0.201	0.031	0.23	6.458	<.001	0.14	0.262	0.868	1.152
Filial Perception	0.193	0.032	0.206	6.057	<.001	0.13	0.255	0.956	1.046

### Mediating effect analysis

Bootstrapped mediation analyses with 5,000 resamples were conducted to examine the indirect associations of dual filial piety with filial practice through parent–child relationship and filial perception. The results are shown in Tables 3 and 4.

**Table 3:** Bootstrapped indirect effects for the reciprocal filial piety model

Path	B	β	SE	95% CI (β)	p
X → M₁ (a₁)	0.092	0.121	0.0264	[0.040, 0.144]	0.001
X → M₂ (a₂)	0.129	0.182	0.0244	[0.081, 0.177]	<.001
M₁ → Y (b₁)	0.200	0.228	0.0292	[0.142, 0.257]	<.001
M₂ → Y (b₂)	0.193	0.206	0.0316	[0.131, 0.255]	<.001
X → Y (c')	0.008	0.012	0.0230	[− 0.037, 0.052]	0.731
X → M₁ → Y	0.018	0.028	0.0086	[0.012, 0.046]	—
X → M₂ → Y	0.025	0.038	0.0099	[0.020, 0.059]	—
Total Indirect Effect	—	0.065	0.013	[0.040, 0.092]	—
Total Effect	0.051	0.077	0.0232	[0.006, 0.097]	—

**Table 4:** Bootstrapped indirect effects for the authoritarian filial piety model

Path	B	β	SE	95% CI (β)	p
X → M₁ (a₁)	− 0.201	− 0.334	0.0198	[− 0.239, − 0.162]	<.001
X → M₂ (a₂)	− 0.031	− 0.056	0.0196	[− 0.070, 0.007]	0.110
M₁ → Y (b₁)	0.200	0.228	0.0308	[0.139, 0.260]	<.001
M₂ → Y (b₂)	0.195	0.208	0.0311	[0.134, 0.256]	<.001
X → Y (c')	− 0.002	− 0.004	0.0185	[− 0.038, 0.034]	0.916
X → M₁ → Y	− 0.040	− 0.076	0.0128	[− 0.103, − 0.052]	—
X → M₂ → Y	− 0.006	− 0.012	0.0081	[− 0.028, 0.003]	—
Total Indirect Effect	—	− 0.088	0.0146	[− 0.117, − 0.060]	—
Total Effect	− 0.048	− 0.092	0.0183	[− 0.084, − 0.012]	—

In the reciprocal filial piety model, the indirect effect through parent–child relationship was significant (a₁ = 0.121, *p* < 0.001; b₁ = 0.228, *p* < 0.001; indirect effect β = 0.028), and the indirect effect through filial perception was also significant (a₂ = 0.182, *p* < 0.001; b₂ = 0.206, *p* < 0.001; indirect effect β = 0.038). The total indirect effect was β = 0.065, 95% CI [0.006, 0.097] (B = 0.051). The direct effect was not significant (c′ = 0.012, *p* = 0.731). The structural equation model showed acceptable fit (χ2/df = 3.196, GFI = 0.953, CFI = 0.905, RMSEA = 0.052).

In the authoritarian filial piety model, the indirect effect through parent–child relationship was significant and negative (a₁ = −0.334, *p* < 0.001; b₁ = 0.228, *p* < 0.001; indirect effect β = −0.076, 95% CI [−0.103, −0.052]). The indirect effect through filial perception was not statistically significant (a₂ × b₂ = −0.012, 95% CI [−0.028, 0.003]). The total indirect effect was β = −0.088, 95% CI [−0.117, −0.060]. The structural equation model also showed acceptable fit (χ2/df = 3.045, GFI = 0.953, CFI = 0.943, RMSEA = 0.050).

## Discussion

This study examined whether dual filial piety was associated with college students’ filial practice through two proposed mediators: parent–child relationship and filial perception. Overall, the results indicate that the observed associations in this sample were primarily indirect rather than direct. In other words, filial piety was linked to filial practice mainly through relational and cognitive pathways after the mediators were taken into account.

### Differential roles of reciprocal and authoritarian filial piety

The findings indicate that reciprocal filial piety and authoritarian filial piety were linked to filial practice in different ways. Reciprocal filial piety was indirectly associated with filial practice through both parent–child relationship and filial perception, whereas authoritarian filial piety was indirectly associated with filial practice mainly through parent–child relationship. These patterns are consistent with the dual filial piety framework, which distinguishes between an emotionally reciprocal dimension and an obligation-oriented dimension. Because the direct effects were not significant, however, the results should be interpreted as evidence of indirect associations rather than of direct causal influence [35].

The non-significant direct effects also help explain why a simple positive-versus-negative interpretation of filial piety is insufficient. In this sample, authoritarian filial piety was not uniformly linked to every aspect of filial practice; rather, its negative association appeared to operate mainly when it coincided with poorer relational experiences. This suggests that relational context matters for understanding when filial norms are translated into behavior [36].

Taken together, these results suggest that, in this sample, filial values were more closely linked to filial practice when they were accompanied by supportive parent–child relationships and stronger filial perception.

### Parent–child relationship as a relational pathway

Parent–child relationship emerged as a significant pathway in both models, but the direction of association differed across the two forms of filial piety. This finding supports the view that relational quality is an important context linking filial values to filial practice. This pattern suggests that a more positive relationship may facilitate the expression of care-related behavior, whereas strained relationships may reduce individuals’ willingness to conduct such behavior [37, 38].

For reciprocal filial piety, the positive indirect path through parent–child relationship suggests that mutual respect, communication, and trust are linked to stronger filial practice. This interpretation is consistent with prior work showing that supportive family interaction can facilitate the internalization and enactment of family responsibilities [39, 40].

For authoritarian filial piety, the negative indirect path through parent–child relationship indicates that higher authoritarian filial piety scores co-occurred with lower parent–child relationship scores, which in turn were associated with lower filial practice scores. This interpretation should not be generalized beyond the present sample, because the study did not test boundary conditions such as family structure, socioeconomic status, or regional differences [41–43].

### Filial perception as a cognitive pathway

Filial perception also functioned as an important pathway, but only for reciprocal filial piety. This finding suggests that reciprocal filial piety may be associated with filial practice partly because it is linked to a clearer and more willingly endorsed understanding of children’s responsibilities toward parents [31, 33].

The significant indirect path from reciprocal filial piety through filial perception is consistent with the idea that mutually respectful family experiences may strengthen responsibility recognition and thereby support practice. In contrast, the non-significant indirect path for authoritarian filial piety suggests that externally emphasized obligation does not necessarily translate into internalized responsibility cognition in the same way [44, 45].

This difference between the two models highlights that not all forms of filial value endorsement operate through the same pathway. In the present study, parent–child relationship was relevant to both models, whereas filial perception was relevant only to the reciprocal filial piety model. In the context of changing intergenerational expectations in contemporary China, this pattern suggests that the translation of filial values into behavior may depend not only on traditional norms [26] but also on the quality of ongoing family interaction and on how young adults interpret filial responsibility [46–55].

## Conclusion

### Key Findings

This study examined two parallel mediation models linking dual filial piety to filial practice among college students.

1. Reciprocal filial piety showed positive indirect associations with filial practice through both filial perception and parent–child relationship.
2. Authoritarian filial piety showed a negative indirect association with filial practice primarily through parent–child relationship.
3. In both models, the direct effects of filial piety on filial practice were not significant after the mediators were included.
4. These findings suggest that filial practice may be linked to dual filial piety mainly through relational and cognitive pathways rather than through direct effects alone.

### Limitations and future directions

First, the cross-sectional design does not permit causal inference. Longitudinal or cross-lagged designs are needed to examine how filial values, relationship quality, and filial practice influence one another over time.

Second, the study relied on self-report measures, which may have introduced shared-method variance and social desirability bias despite the procedural and statistical checks that were used.

Third, the sample was a convenience sample of college students from one region of China. The findings therefore should not be generalized uncritically to other age groups, occupational groups, or cultural and regional contexts.

Fourth, the average variance extracted (AVE) of the reciprocal filial piety scale was 0.42, slightly below the conventional threshold of 0.50. Future studies may use more items or a revised version of the scale to further improve convergent validity.

Fifth, the measure of parent–child relationship was adapted from an attachment-based instrument; future studies may benefit from including additional relationship-quality indicators and more contemporary dimensions of filial interaction.

Overall, the present study provides initial cross-sectional evidence that reciprocal and authoritarian filial piety are associated with filial practice through partly different relational and cognitive pathways among college students in contemporary China. This pattern may help explain how filial values are translated into behavior under changing intergenerational expectations, but it should be interpreted cautiously and tested further with more diverse samples, stronger designs, and more fine-grained measures.

## Data Availability

The data presented in this study are available on request from the corresponding author. Ph. D Candidate Ge Zengming mail gezengming2024@163.com.
